# The Health Extension Program and Its Association with Change in Utilization of Selected Maternal Health Services in Tigray Region, Ethiopia: A Segmented Linear Regression Analysis

**DOI:** 10.1371/journal.pone.0131195

**Published:** 2015-07-28

**Authors:** Tesfay Gebregzabher Gebrehiwot, Miguel San Sebastian, Kerstin Edin, Isabel Goicolea

**Affiliations:** 1 Department of Public Health, College of Health Sciences, Mekelle University, Mekelle, Ethiopia; 2 Department of Public Health and Clinical Medicine, Epidemiology and Global Health, Umeå University, Umeå, Sweden; 3 Department of Nursing, Umeå University, Umeå, Sweden; Brighton and Sussex Medical School, UNITED KINGDOM

## Abstract

**Background:**

In 2003, the Ethiopian Ministry of Health established the Health Extension Program (HEP), with the goal of improving access to health care and health promotion activities in rural areas of the country. This paper aims to assess the association of the HEP with improved utilization of maternal health services in Northern Ethiopia using institution-based retrospective data.

**Methods:**

Average quarterly total attendances for antenatal care (ANC), delivery care (DC) and post-natal care (PNC) at health posts and health care centres were studied from 2002 to 2012. Regression analysis was applied to two models to assess whether trends were statistically significant. One model was used to estimate the level and trend changes associated with the immediate period of intervention, while changes related to the post-intervention period were estimated by the other.

**Results:**

The total number of consultations for ANC, DC and PNC increased constantly, particularly after the late-intervention period. Increases were higher for ANC and PNC at health post level and for DC at health centres. A positive statistically significant upward trend was found for DC and PNC in all facilities (p<0.01). The positive trend was also present in ANC at health centres (p = 0.04), but not at health posts.

**Conclusion:**

Our findings revealed an increase in the use of antenatal, delivery and post-natal care after the introduction of the HEP. We are aware that other factors, that we could not control for, might be explaining that increase. The figures for DC and PNC are however low and more needs to be done in order to increase the access to the health care system as well as the demand for these services by the population. Strengthening of the health information system in the region needs also to be prioritized.

## Introduction

Securing pleasant and safe motherhood for both mother and child is a fundamental reproductive health right [1, 2]. While the attention given to maternal health care services has increased in the past years, inequalities in access to these services remain prominent [3, 4]. As a result, over 300,000 women worldwide, and more than 162,000 women in Sub-Saharan African (SSA) countries, still die due to the process of pregnancy and child birth [5–8].

Ethiopia is a major contributor to this death toll, with a maternal mortality ratio of 676 per 100,000 live births [9, 10]. One way to achieve women’s reproductive health rights and to tackle maternal mortality is by strengthening and ensuring the transformation of the entirety of all health systems for better health service delivery in ways that make them more accessible, available, people-centred, safe and effective, and better quality [11].

In its 2008 report, the World Health Organization (WHO) emphasized the importance of revitalizing primary health care, refocusing to strengthen the health system through four reforms, namely: 1) universal coverage; 2) primary care; 3) public policy; and 4) leadership. It is widely recognized that governments need to be determined in developing a coordinated, efficient and comprehensive health system to promote primary care and universal coverage if the health of the population is to be improved [11–15].

Responding to this interest, the Ethiopian Ministry of Health committed to launching a community-based health care system in 2003 – the Health Extension Program (HEP), rooted in a primary health care approach [16–17]. The HEP is an innovative programme designed to improve equitable access to preventive essential health interventions through community-based health services and to achieve significant basic health care coverage. The programme is undertaken through the provision of a staffed health post to serve an area of approximately 3,000 to 5,000 people – a *kebele*, the lowest administrative unit. Each *kebele* has one health post and two health extension workers (HEWs). The HEWs, who are the first point of contact of the community in the health system, are employed after completion of one year’s training [18, 19]. The HEP has been implemented throughout Ethiopia, with more than 33,000 HEWs trained and deployed since 2004. In rural areas, the HEP is composed of 16 intervention packages categorized into four areas: “hygiene and environmental sanitation” (seven packages), “family health” (five packages), “disease prevention and control” (three packages) and “health education and communication” (one package). All components of maternal and child health services are free of charge throughout the whole health care system [20].

Despite strong efforts in spreading the HEP throughout the country, only a few studies have assessed the impact of the HEP, but these have shown positive increases in some of its sub-components such as community participation, community health awareness, immunization coverage and hygiene and sanitation [21–22]. Other aspects of the HEP, like the access to institutional delivery, remain poor. According to the national health sector development programme report of 2011–2012 (HSDP IV), the prevalence of skilled delivery attendance is only 18.4%. Better figures have been achieved for family planning, antenatal care and post-natal care (68%, 56.2% and 34% respectively). All these data are worst in urban poor and rural areas. So, in terms of maternal health, much remains to be done to achieve the Millennium Development Goal 5 (MDG 5), particularly when it comes to skilled delivery attendance and post-natal care. The phases of HEP are indicated as milestones in pages 5–6.

Despite the massive support and recognition the programme has received, both nationally and internationally, no evaluation of its impact on access to services has been documented [21–22]. This paper aims to assess the association of the HEP with improved utilization of maternal health services—mainly antenatal care (ANC), delivery care (DC) and post-natal care (PNC) —in Northern Ethiopia using institution-based retrospective data over a period of 10 years (2002–2012)".

### HEP milestones

In year 2001–2002, a one-month workshop led by the former RHB head was conducted attending thirty five district health officers and Tigray health bureau staffs. They developed six guidelines including a HEP manual which were distributed to all primary health care units. Afterwards the HEP was launched in 2003 at national level. Construction of health posts in Tigray begun in 2002 and underwent until 2004/2005, over 500 health posts were built. The health posts of the study districts were built in 2004. Health posts were equipped and furnished during 2004–2005 by the government in collaboration with UN bodies. More than 1200 HEWs were trained and deployed in two consecutive years, 2005–2006; each health post of the study district was staffed by one HEW in 2005. HEP supervisors were also assigned. The HEWs were later mentored about midwifery services for one month at health centres and hospitals under supervision of nurses and midwives.

The HEWs were, immediately after they were deployed, engaged in providing training about the HEP components to community members. Participants who were considered to have extra ordinary performance of other developmental activities were selected as model families. Afterwards, the model families were retrained (2005/2006) as volunteer community health workers (VCHWs) and community health promoters (CHPs) and were organized to work jointly with the HEWs.

As part of strengthening the HEP, integrated refresher training on health service delivery was provided in 2007. Training of trainers was conducted for 105 supervisors at regional level, and afterwards it was cascaded to all HEWs in 35 districts. The various implementations of HEP phases at all levels were financed through the resources that were contributed by the government, international donors and local communities [21].

In year 2007, intensive advocacy and mobilization for policy makers was conducted at four zones of the region. Various sector heads (such as education, agriculture, women affairs office, executive administration of the district), and community leaders participated in the forum.

Integrated community case management for under five children has been provided since 2007/08. Similar to other districts in the region, transportation services through modern ambulances for labouring women in the study districts started in 2009/10. Moreover, a new initiative of using traditional ambulances by community-organized efforts has been practiced since 2012/13. The health development army was also established in 2011–2012. To improve the health management information system, a family folder that records the status of household members in the family, has been applied in the study districts since 2011–2012.

## Methods

### Study area

Tigray regional state is located in the northern part of the country and has an estimated total population of 4.3 million, of whom 50.8% are female. Around 80% of the population live in rural areas and the majority of the inhabitants are orthodox Christians [23]. The region is divided into seven zones and 47 districts *(weredas)*, of which 35 are rural. There is one specialized referral hospital in the region, as well as five zonal hospitals, seven district hospitals, 208 health centres (HCs) and more than 600 health posts (HPs). Coverage estimates from the Tigray Health Bureau (THB) indicate 75% for the first visit of ANC, 32% for skilled delivery (those attended by a nurse, midwife, health officer [non-physician clinician] or a physician), 13% for clean and safe delivery (those attended by HEWs), 51% for PNC (women who, after delivery, are assisted at health facilities by nurses/midwives or visited by HEWs at households) and 90% for family planning use [24].

The HEP was first implemented in Tigray in the middle of 2005. New health posts were built and at least one HEW was assigned to each health post. HEWs’ tasks included delivering basic curative services at the health post and promotive and preventive services in the community. HEWs may also attend deliveries, although if a complication emerges they have to refer to the health centre, which might be two to three hours’ walking distance. However, nowadays, it is mainly recommended that childbirth in Tigray should occur at least at health centre level, despite the fact that this is inaccessible to most rural women.

Based on the HEP guidelines, essential supplies have been delivered to health posts (Table 1).

**Table 1:** List of supplies delivered at health posts.

Ser. No.	Purpose of supplies	Items
1	Health promotion service	Educational materials (in local language) Posters and pamphlets, guidelines and working manuals
2	Preventive services	Delivery couch, phetoscope, vaccine carrier, cold box, height measuring board, adult weighing scale, child weighing scale, oral contraceptives, injectables and condoms, family planning, registration book
3	Basic curative services	Examination bed, stethetoscope, sphygmomanometer, rapid test kits (for malaria), fansidar tablets (for malaria), oral rehydration salts, tetracycline eye ointment tubes, iron tablets, outpatient and antenatal, expanded program of immunization (EPI) and delivery care registration books

Since maternal health was one of the major components of the HEP packages, capacity-building activities have been performed periodically. After the program starts to deliver the promotive and preventive health services, major efforts have been carried out to refill supplies, equipment and materials. Occasionally, refresher training on clean and safe delivery has also been provided to HEWs.

### Selection of districts

Prior to data collection, a situational analysis was carried out to assess the availability of data through reviewing health profiles and reports and conducting discussions with concerned officials. Of the 34 rural district health offices, six were selected based on geographical distribution.

When visiting the districts, documents were reviewed for completeness of the data based on a checklist prepared to answer the research question. Too many missing data was found in three of the districts and these were excluded. Due to the limited resources it was not possible to replace the excluded districts.

The study districts Kilte-Awlaelo, Ganta-Afeshum and Hintalowajirat, which are located in the eastern, north-eastern and south-eastern parts of the state of Tigray, respectively (45, 120 and 35 kilometres from the capital Mekelle, respectively) were finally selected.

### Data collection

The variables in the checklist included the name of the district and health facility, date, facility code, total population of the catchment area and type of services provided, namely: 1) antenatal care, defined as the number of women who attended a HP or HC at least once during their pregnancy; 2) delivery care, meaning the number of women assisted by skilled health personnel at health centres and clean and safe deliveries by HEWs at health posts; and 3) post-natal care, included the number of women checked by a health worker at a HC or by an HEW at a HP or during a household visit at least once during the 45-day period after delivery.

The format of the registration book and the reporting template was checked for its compliance to each other, where both of the records were found to accommodate the checkups of ANC and PNC from first visit till four which possibly avoids double counting of women. Two pairs of people at each district carried out the data collection process. From the district health office, the person who was in charge of the archive office and the maternal health expert were fully engaged in sorting the relevant documents and handing over to the PI. Then, the first author and a MPH student were involved in labelling and recording the data after a proper check.

### Data analysis

The data from the period between July 2002 and June 2012 was extracted from the district offices and checked for accuracy, cleaned in an Excel spreadsheet and then exported to the Stata software for analysis (clean and raw data can be found S1 Table, S2 Table, S3 Table and S4 Table). In total, data from 16 health centres and 45 health posts was collected.

The 10-year data of the three outcome variables (antenatal care, delivery and post-natal care) was aggregated on a quarterly basis. The data was analyzed separately by HC, by HP and as total. The period was subdivided into three phases, named as pre-intervention (July 2002–June 2005), immediate intervention (July 2005–June 2008) and late-intervention (after July 2008).

The pre intervention phase shows the period when the program had not yet started. The proximity of health posts and the presence of HEWs were not ensured. The promotive and preventive services were neither provided at the health posts nor at household level. The data source for the period of the pre-intervention was from poorly designed health posts (built by the community) that were staffed by primary health care workers and primary midwifes who trained for six months after they accomplished high school and from few health centres and moderate number of poorly renovated clinics with less number of human resources. Immediate intervention was defined as when the HEP was initiated and late-intervention as when the HEP was consolidated in the region. The year 2005 was selected as the starting point of the intervention because of two reasons: 1) the minimum essential supplies were ensured at health posts, and 2) the health extension workers started to provide promotive and preventive services at health posts and households that year.

Data were analyzed using segmented linear regression. This technique was applied to control for secular trends and to adjust for potential serial correlation of the data [25–27].

The linear regression can be represented as an equation:

Yt=B0+B1*time+B2*intervention+B3*postslope+et

Where Yt is the outcome variable at time t and time is a continuous variable indicating the period from the beginning of the study up to the end of the observation. The intervention variable was coded 0 for the pre-intervention time (from July 2002-June 2005) and 1 for the post-intervention time (July 2005-June 2012). The post-slope variable was coded 0 up to the last point of the pre-intervention period (till June 2005) and coded from 1 onwards sequentially after the intervention started. In this model, B0 captures the baseline level of the outcome at time 0 (beginning of the period); B1 estimates the structural trend in utilization, independently from the intervention; B2 estimates the immediate impact of the intervention or the change in level in the outcome of interest after the intervention; B3 reflects the change in trend after the intervention; and *et* reflects the residual error of the calculated value.

During the analysis two models were developed. Model 1 was used to estimate the level and trend changes associated with the immediate period of intervention (after July 2005). Changes related to the late-intervention period (after July 2008) were estimated by Model 2. Since autocorrelation is commonly detected in longitudinal data, the Prais–Winsten estimator was applied [25].

### Ethical considerations

The study received ethical approval from the University of Mekelle, Health, Research Ethical Review Committee of the College of Health Sciences, Northern Ethiopia (approval letter ERC 0122/2012). A letter of permission was issued from the Tigray Regional Health Bureau and the district health authorities. Written informed consent from the archive officers and maternal health experts was obtained. The records was anonymized and deidentified prior to analysis.

## Results

### Trends in attendances in health centres and health posts

**Fig 1:**
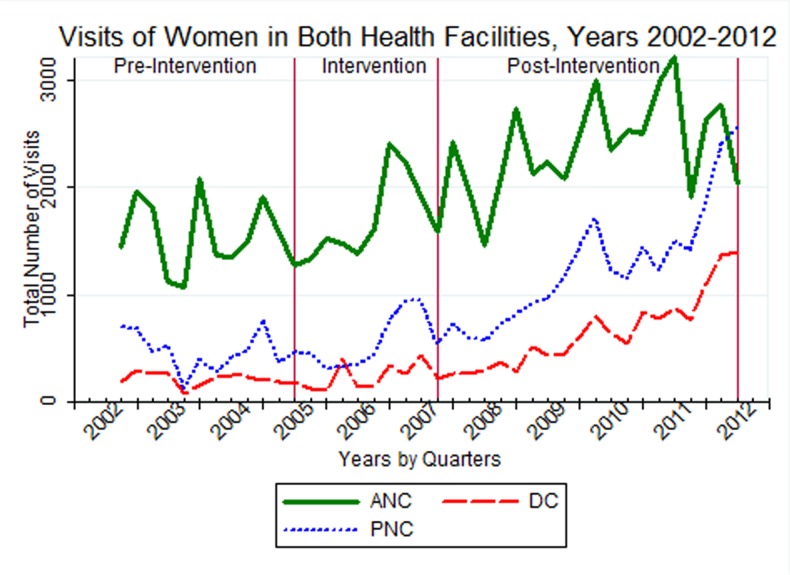
Trends of antenatal, delivery and post-natal care attendants at health centres and health posts (n = 61) from July 2002 to June 2012 in selected districts of Tigray region, Ethiopia.

From one year after the intervention initiated, the number of women attending health centres (HCs) and health posts (HPs) for the three types of services showed an increasing trend in the consecutive years. The data showed a sharp rise of users flow in ANC services from July 2007 to July 2011, decreasing in the last year of the study. An increasing trend, particularly from July 2008, was observed for DC. PNC utilization fluctuated over the years of the study, with a sharp increase from July 2011 to June 2012 (Fig 1).

### Trends in attendances at health centres

**Fig 2:**
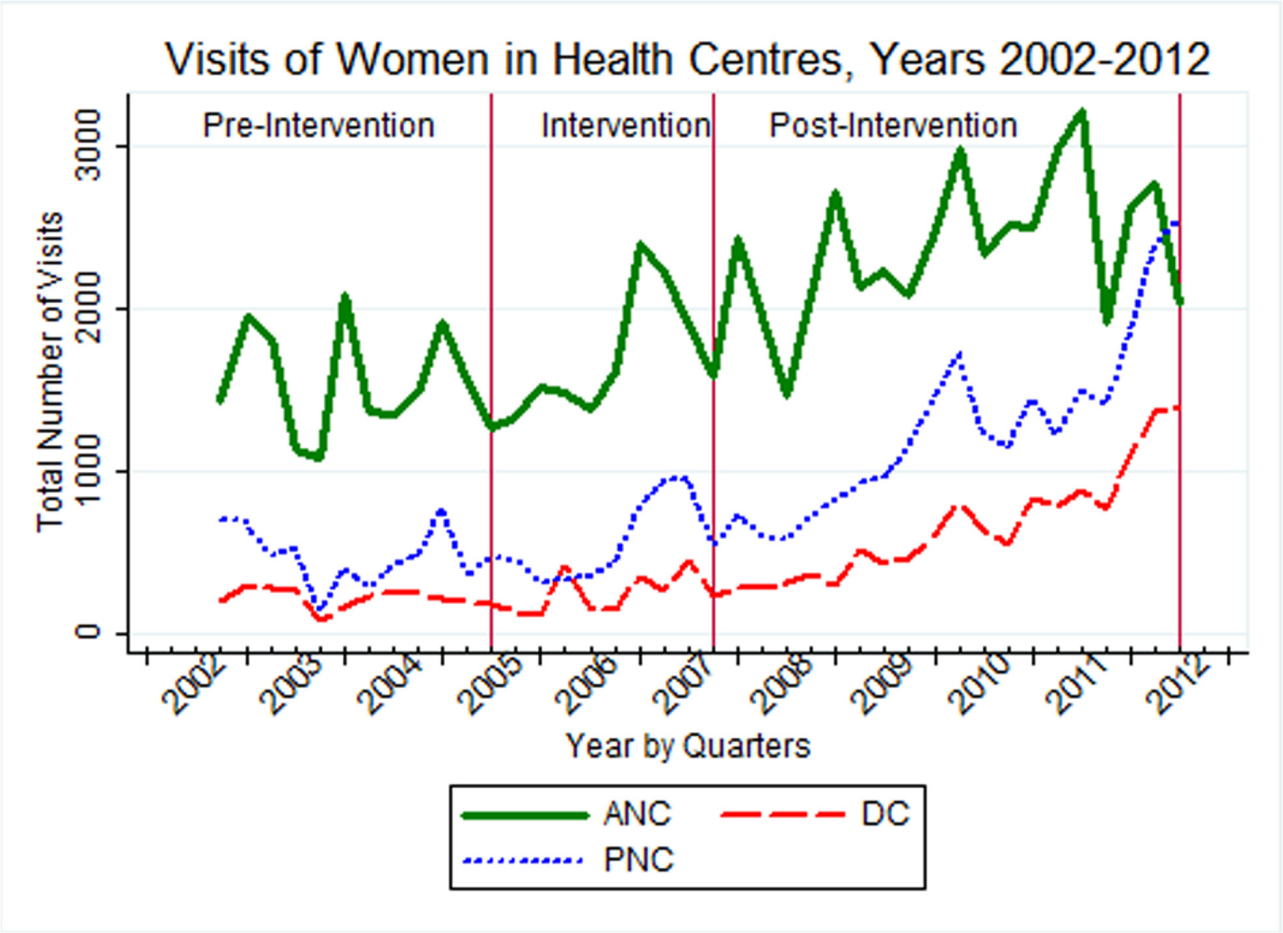
Trends of antenatal, delivery and post-natal care attendants at health centres (n = 16) from July 2002 to June 2012 in selected districts of Tigray region, Ethiopia.

At health centre level, the total number of ANC users (47,724) in the whole period was between 4.9 to 3.3 times higher when compared with women who were assisted by skilled health attendants for delivery (9,684) and received post-natal care services (14,311), respectively. In the years before the intervention, a downward trend of the services was observed. However, there was a recovery in the number of ANC visits and a constant increase in the number of skilled attendant deliveries and PNC visits after the implementation stage (Fig 2).

### Trends in attendance at health posts

**Fig 3:**
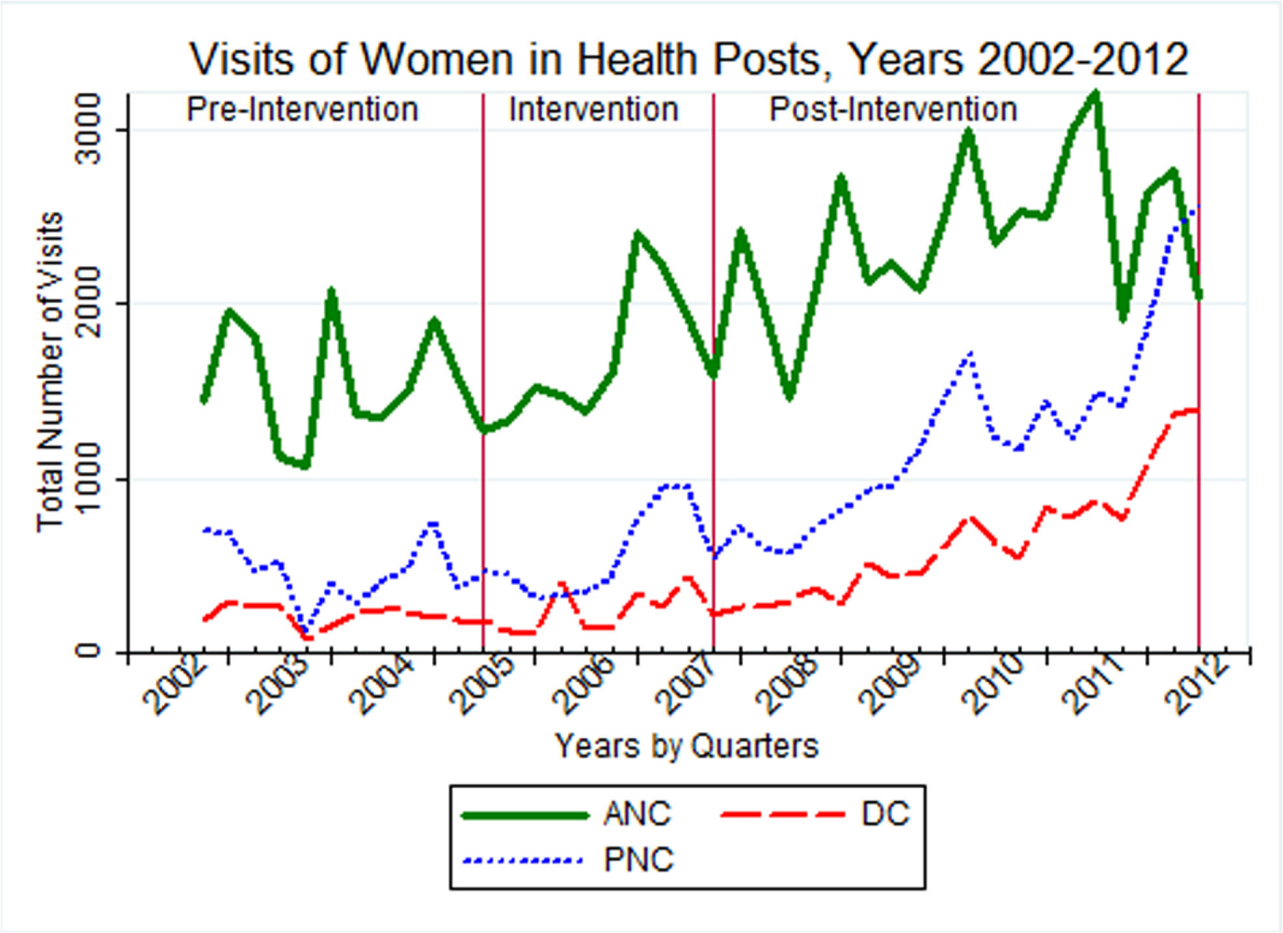
Trends of antenatal, delivery and post-natal care attendants at health posts (n = 45) from July 2002 to June 2012 in selected districts of Tigray region, Ethiopia.

In the pre-intervention phase, a small but gradual increase in the number of maternal health attendances was observed. Soon after the HEP started, the trend of user flow increased in all types of services in a consistent manner. During the whole period, the total number of women receiving ANC (31,697) and PNC (21,033) services was between 4 and 2.7 that of women who received clean and safe delivery (7,825). In the late-implementation period (July 2008–June 2012), the number of women visiting ANC services stabilized, while the number of women who received delivery and PNC continued to increase regularly (Fig 3).

### Regression analysis

The results from Model 1 highlighted that at the beginning of the period there were on average 1652.38 and 18.13 visits to ANC services at health centres and health posts, respectively. The number of consultations increased after the immediate intervention by 116.88 visits per quartile at HPs, though a decrement of −39.14 was observed at HCs; none of them was statistically significant (Model 1). However, a significant change in trend after the intervention was observed in health centres (p = 0.009) but not in health posts (p = 0.76) (Table 2).

**Table 2:** Model 1 regression analysis (corrected for auto correlation) of attendants for ANC, DC, PNC at health centres, health posts and both, July 2002–June 2012.

		B0	B1 (time)	B2 (intervention)	B3 (post slope)
ANC	Total	1667.53	-17.15 (0.62)	75.14 (0.76)	61.36 (0.09)
	HC	1652.38	-54.69 (0.02)	-39.14 (0.82)	68.42 (0.009)
	HP	18.13	37.26 (0.10)	116.88 (0.46)	-7.05 (0.76)
Delivery care	Total	387.78	-26.62 (0.43)	-108.73 (0.41)	57.48 (0.12)
	HC	452.99	-36.57 (0.32)	-45.63 (0.68)	60.37 (0.13)
	HP	-26.72	10.45 (0.05)	-115.23 (0.004)	8.69 (0.11)
Postnatal care	Total	-10083.82	404.10 (0.21)	48.02 (0.88)	-177.71 (0.43)
	HC	467.59	-32.03 (0.38)	62.91 (0.63)	49.52 (0.21)
	HP	-86.84	28.39 (0.22)	-254.04 (0.05)	20.58 (0.40)

P values are in brackets.

During the late-implementation period (Model 2), the estimated mean number of ANC consultations increased significantly, by 122.75 and 326.47 visits per quartile at health centres (p = 0.05) and health posts (p = 0.006) respectively. There was a positive change in trend observed at the health centres (p = 0.04), while a negative one was seen at the health posts (p = 0.01) (Table 3).

**Table 3:** Model 2 regression analysis (corrected for auto correlation) of attendants for ANC, DC, PNC at health centres, health posts and both, July 2002−June 2012.

		B0	B1 (time)	B2 (intervention)	B3 (post slope)
ANC	Total	1412.00	19.50 (0.11)	456.782 (0.07)	-2.74 (0.90)
	HC	1400.04	-18.70 (0.04)	122.75 (0.05)	37.18 (0.04)
	HP	9.85	38.41 (0.00)	326.47 (0.006)	-39.54 (0.01)
Delivery care	Total	321.62	-15.82 (0.52)	-160.97 (0.21)	54.50 (0.038)
	HC	208.56	-3.67 (0.46)	-139.03 (0.11)	49.60 (0.000)
	HP	-6.83	6.18 (0.001)	91.55 (0.01)	11.46 (0.001)
Postnatal care	Total	315.37	14.08 (0.28)	-41.19 (0.84)	84.38 (0.002)
	HC	333.10	-4.03 (6.0)	-148.88 (0.21)	45.65 (0.003)
	HP	-21.92	18.50 (0.01)	107.78 (0.37)	37.26 (0.007)

P values are in brackets.

Prior to the intervention, there was an average of 452.99 visits for skilled delivery attendants at health centres (HCs) and −26.72 visits for clean delivery (HPs). Soon after the immediate intervention (Model 1), while a decrement in consultations per quartile was observed in both health centres and health posts, a non-significant increase occurred in the trend (Table 2). In the late-implementation period (Model 2), a significant positive change was observed at the health post levels (p = 0.01) just after the intervention and in the trend for both (p<0.01) health centres and health posts (Table 3).

Average visits of PNC attendants prior to the intervention were 467.59 and −86.84 at health centres and health posts respectively. Right after the intervention was implemented, the number of consultations at health posts decreased significantly. Despite the increased number of attendants at health centres and health posts, a non-significant change of trend was observed (Model 1, Table 2). During the late-intervention period, decreases in the average number of consultations at both health centres and health posts were compensated by positively significant changes in an upward trend (Model 2, Table 3).

## Discussion

Overall, our findings indicated a positive impact of the HEP in the use of maternal health care services. The total number of consultations for ANC, DC and PNC increased constantly, particularly after the late-intervention period. A difference in trends between HCs and HPs of ANC users was observed during the whole period, whereas a similar trend for delivery care and PNC visits was observed in both (HCs and HPs). A positive statistically significant trend was found for DC and PNC in HCs and HPs. The positive trend was also present in ANC at health centres, but not at health posts.

### Antenatal care

In the present study, the substantial overall increase of women receiving ANC was due to the increased number of users to HPs and health centres. Similar to our findings, an intervention study of HEP in 2009 revealed a huge flow of pregnant women using ANC from the intervention group compared to the control group [21]. Three possible reasons could be discussed: 1) the proximity of health posts to the community; 2) women’s increased perception of improved quality of health workers and health facilities; and 3) the close attention facilitated by female health workers.

Proximity to health facilities has been reported as a major predictor of ANC utilization in the literature [28–34]. In the recent past, the Tigray Health Bureau and the rural communities in collaboration with Federal Ministry of Health (FMOH) have made great effort to ensure that all districts are covered by one health post and two HEWs in each *kebele*. This closeness facilitates access for pregnant women to attend ANC checkups [20, 22, 35–37].

In order to strengthen the HPs, the THB has also regularly delivered training courses to health workers and consistently replenishes the facilities’ supplies. These factors might have contributed to increasing the perceived quality of the ANC services [10, 22, 35].

From the outset, the HEP has trained only female workers [17–18]. The main reason for this was to create a trustful environment for women in the area of reproductive health. Feelings of safety and privacy might have facilitated the demand for ANC services. The process of identifying pregnant women through house-to-house visits by the HEWs in collaboration with the volunteer community health workers could also be an additional reason that might have influenced women to decide to seek care during pregnancy. The frequent visits of households by the HEWs, the possession of a family folder and being a model family have been reported as factors, which improve access to ANC services [10, 22].

More work needs to be done regarding reasons for not using ANC in the region. Lack of awareness of ANC benefits, feeling shame, the influence of husband’s approval, excessive workload at HPs, poor quality of care and distance have been reported as explanations in previous studies [16, 37–42].

### Delivery care

An increase in the number of institutional delivery was observed in both HCs and HPs during the late-intervention period, particularly in the case of HCs. Improved perception of the advantages of institutional delivery such as feeling safe and better hygiene, and the determined effort of HEWs in providing maternal health education, might be factors contributing to the increase. Apart from the overall improvements of infrastructure and standard of living of the society, ways in which the HEP might have contributed to expand the access to facility-based DC include: 1) the efforts of the FMOH in bringing the agenda of institutional delivery as a priority and working hand in hand with its partners, 2) the commitments of THB, and implementing partners in paying a great attention to improve access to institutional delivery, and 3) the endeavor of the local government to introduce transport access for women on labour (through organized community effort) and the 24-hours service policy of health centres and hospitals.

Despite all the efforts, the recent EDHS report has shown a prevalence of 24.7% (for the region), whereas, data from the THB for all the districts are higher (32.2%) [35, 43], but still far from the national target of 62% for the year 2014/15.

Cultural and religious traditions, together with family opinions, play a major role in the decision-making process regarding where to deliver in Tigray. Moreover, women in the region have also complained regarding issues of quality of care, unskilled health workers and unfriendly staff attitudes, services’ availability and limited access to transportation, at both HCs and HPs [36–37, 42–46]. Besides, results from a recent local study indicate the absence of water and electricity at health posts, which has been mentioned by HEWs and midwives as barriers for institutional delivery [47]. Quantitative surveys have also pointed to health workers’ poor counselling of pregnant women during ANC visits at health facilities as a reason for low use [36–37, 48–49].

### Post-natal care

An important increase in post-natal care was observed after the late-intervention period, particularly at HP level. Consistent to our finding, a large study conducted in 101 rural districts of the country have shown the association between the HEP intensity and increased users of PNC service [50]. Some of the reasons mentioned in the case of ANC could also be relevant in this case, such as the preventive work that HEWs conduct both at health posts and in the community, the proximity of the service to the household and the attention given by female workers.

The sharp increase reported from July 2011 onwards could be the result of a greater focus of the HEP in raising awareness at community level of the importance of the PNC attention.

### Limitations

Statistics from the three districts initially selected could not be used. Reasons need to be further explored, but could be attributed to inadequate data collection and reporting from the HPs and HCs or inadequate data entry at the district level. This calls for the need to strengthen the organization and quality of the health information system in the region. Use of retrospective data might be questionable for its validity. In these setting validity of these data might be hindered by poor handling of the archives and an absence of electronic medical recording system in the facilities. This could have affected the interpretation of the results in either underestimating or double counting of users in the study. However, an effort was made to follow the completeness and accuracy of the data by cross checking the reports submitted from HCs and HPs to the districts by the PI and the MPH student. While we acknowledge that certain incompleteness could still remain, these inaccuracies might have been improved after the implementation of the family folder at health posts.

Some methodological issues should also be mentioned. An advantage of the design was the use of multiple observations over a long period, before and after the intervention, which enabled us to control for pre-existing trends and study the dynamics of the changes in response to the intervention. In this type of designs, a general recommendation is to include at least 12 data points before and 12 after the intervention – this was fulfilled in this study [25]. However, we are aware that this may not necessarily detect the causal determinants of that impact.

Explanation for changes could not be due to the HEP only, but to other external factors that also happened during those years such as: increase in per capita income, building roads and infrastructure, strengthening micro and small scale enterprises which reduces the trend of unemployment, expansion of agricultural programs and increased access to education. Because of unavailability of data, the external factors influencing the outcomes are not controlled. Due to the fact that our sample was taken from only a small amount of districts, the issue of generalizability needs to be carefully handled.

## Conclusion

Our findings reveal that there has been an overall increase in ANC, DC and PNC use after the introduction of the HEP, particularly in ANC and PNC at health post level and DC at health centres. We are aware that other factors, that we could not control for, might be explaining that increase. The figures for DC and PNC are however low and more needs to be done in order to increase the access to the health care system, as well as the demand for these services by the population. Strengthening of the health information system in the region also needs to be prioritized. The THB is currently implementing several interventions such as the use of ambulances and mobile phones for referrals and training programs for improving the quality of care that will hopefully contribute to increase maternal health coverage in the nearest future.

## Supporting Information

S1 Table Clean data set on antenatal care delivery care and postnatal care visits in three districts. (XLSX)

S2 Table Raw data set of antenatal, delivery care and postnatal care visits in Ganta-Afeshum. (XLS)

S3 Table Raw data set of antenatal, delivery care and postnatal care visits in Kilte-Awlaelo. (XLS)

S4 Table Raw data set of antenatal, delivery care and postnatal care visits in Hintalo-Wajirat. (XLS)
